# A Comprehensive Review on Water Quality Parameters Estimation Using Remote Sensing Techniques

**DOI:** 10.3390/s16081298

**Published:** 2016-08-16

**Authors:** Mohammad Haji Gholizadeh, Assefa M. Melesse, Lakshmi Reddi

**Affiliations:** 1 Department of Civil Engineering, Florida International University, Miami, FL 33174, USA; mhaji002@fiu.edu (M.H.G.); lreddi@fiu.edu (L.R.); 2 Department of Earth and Environment, Florida International University, Miami, FL 33199, USA

**Keywords:** remote sensing, spaceborne sensors, airborne sensors, water quality indicators

## Abstract

Remotely sensed data can reinforce the abilities of water resources researchers and decision makers to monitor waterbodies more effectively. Remote sensing techniques have been widely used to measure the qualitative parameters of waterbodies (i.e., suspended sediments, colored dissolved organic matter (CDOM), chlorophyll-*a*, and pollutants). A large number of different sensors on board various satellites and other platforms, such as airplanes, are currently used to measure the amount of radiation at different wavelengths reflected from the water’s surface. In this review paper, various properties (spectral, spatial and temporal, etc.) of the more commonly employed spaceborne and airborne sensors are tabulated to be used as a sensor selection guide. Furthermore, this paper investigates the commonly used approaches and sensors employed in evaluating and quantifying the eleven water quality parameters. The parameters include: chlorophyll-*a* (chl-*a*), colored dissolved organic matters (CDOM), Secchi disk depth (SDD), turbidity, total suspended sediments (TSS), water temperature (WT), total phosphorus (TP), sea surface salinity (SSS), dissolved oxygen (DO), biochemical oxygen demand (BOD) and chemical oxygen demand (COD).

## 1. Introduction

Over 40% of the world’s population lives in coastal regions and lake or river shores [1], and this proportion is increasing. The coastal area of rivers and other waterbodies are among the most sensitive environments. Any changes in these fragile ecosystems due to anthropogenic activities can endanger the habitats of fish and other aquatic organisms. Similarly, the need for sustainable urban water supplies requires that the quality of existing available water resources as well as their watersheds need to be under continuous monitoring. Besides, the level of treatment required for human consumption, agriculture, animal husbandry and industry necessitates an understanding of the quality of source waters. In this way, at the beginning of the twentieth century, the importance of water quality has to be considered more than ever, and the concentration of chemicals in sewage and industrial discharges in waterbodies needs to be taken under more precise control [2,3].

Water quality indicators including physical, chemical, and biological properties are traditionally determined by collecting samples from the field and then analysing the samples in the laboratory. Although this in-situ measurement offers high accuracy, it is a labour intensive and time consuming process, and hence it is not feasible to provide a simultaneous water quality database on a regional scale [4,5]. Moreover, conventional point sampling methods are not easily able to identify the spatial or temporal variations in water quality which is vital for comprehensive assessment and management of waterbodies. Therefore, these difficulties of successive and integrated sampling become a significant obstacle to the monitoring and management of water quality.

With advances in space science and the increasing use of computer applications and increased computing powers over recent decades, remote sensing techniques have become useful tools to achieve this goal. Remote sensing techniques make it possible to monitor and identify large scale regions and waterbodies that suffer from qualitative problems in a more effective and efficient manner. The collection of remotely sensed data occurs in digital form and therefore is easily readable in computer processing. Remote sensing techniques have been in use since the 1970’s and continue to be widely used in water quality assessment in the contemporary world [6,7,8,9,10,11,12,13,14,15,16,17,18,19,20,21].

Different sensors mounted on satellites and other platforms, such as aeroplanes, measure the amount of radiation at various wavelengths reflected from the water’s surface. These reflections can be used directly or indirectly to detect different water quality indicators, such as total suspended solids (TSS), chlorophyll-*a* concentration, turbidity, salinity, total phosphorus (TP), Secchi disk depth (SDD), Temperature, pH, Dissolved Organic Carbon (DOC),etc. The spectral characteristics of water and pollutants, which are functions of the hydrological, biological and chemical characteristics of water, etc. [19], are essential factors in the monitoring and assessment of water quality. The study thus introduces the widely employed spaceborne and airborne sensors in water quality investigations and discusses the utility of remotely sensed techniques in the qualitative assessment of waterbodies. Various properties (spectral, spatial and temporal, etc.) of spaceborne and airborne sensors are tabulated to be used as a sensor selection guide. Finally, based on the literature survey, the study presents a compilation of the various sensors useful in the study of some measurable water quality parameters, and investigates in more detail eleven water quality parameters based on the employed approaches to measuring their concentrations.

## 2. An Overview of Water Quality Assessment and Remote Sensing

In-situ data collections are only able to represent point estimations of the quality of water conditions in time and space, and obtaining spatial and temporal variations of quality indices in large waterbodies is almost impossible [18]. Briefly listed below are the most important limitations associated with conventional methods:

1. In-situ sampling and measurements of water quality parameters are labor intensive, time consuming, and costly.
2. Investigation of the spatial and temporal variations and water quality trends in large waterbodies is almost impossible.
3. Monitoring, forecasting, and management of entire waterbodies might be inaccessible, for example due to the topographic situation.
4. Accuracy and precision of collected in-situ data can be questionable due to both field-sampling error and laboratory error.

To overcome these limitations, the use of remote sensing in water quality assessment can be a useful tool. For more than four decades, remote sensing has illustrated strong capabilities to monitor and evaluate the quality of inland waters. Many researchers frequently use the visible and near infrared bands of the solar spectrum (mostly from blue to near infrared region) in their investigations to obtain robust correlations between water column reflection (in some cases emission) and physical and biogeochemical constituents, such as transparency, chlorophyll concentration (phytoplankton), and the organic matters and mineral suspended sediments in different waterbodies [9,10,18,22,23,24,25,26,27,28,29,30]. Although the capabilities of remote sensing to assess water quality are undeniable, this technique alone is not sufficiently precise and must be used in conjunction with traditional sampling methods and field surveying. In other words, to obtain a better insight, an integrated use of remote sensing, in-situ measurements and computer water quality modelling may lead to an increased knowledge of the water quality of water systems. Collaboration between different governmental, federal and private agencies and data sharing is also helpful to increase the data required for regional studies. Kallio [31] has mentioned four advantages of applying remote sensing in compliance with other water quality monitoring programs as below:

1. Gives a synoptic view of the entire waterbody for more effective monitoring of the spatial and temporal variation.
2. Makes it possible to have a synchronized view of the water quality in a group of lakes over a vast region.
3. Provides a comprehensive historical record of water quality in an area and represents trends over time.
4. Prioritizes sampling locations and field surveying times.

Optically active constituents of water that interact with light and change the energy spectrum of reflected solar radiation from waterbodies can be measured using remote sensing [18]. The components, already enumerated in the first section, constitute the majority of important water quality parameters in surface waters. Other parameters include acidity, chemicals, and pathogens, which do not change the spectral properties of reflected light and have no directly-detectable signals, but which may be interpretable and inferable from those detectable water quality parameters with which strong correlations can be found [18,31].

## 3. Spaceborne and Airborne Sensors for Water Quality Studies

Observing sensors are divided into two main categories based on the platforms on which they are situated. Airborne sensors are those that are mounted on a platform within the Earth’s atmosphere (i.e., a boat, a balloon, a helicopter, or an aircraft), and spaceborne sensors are carried by a spacecraft or satellite to locations outside of the Earth’s atmosphere. Understanding the properties of these sensors is necessary to choose an appropriate sensor for the objectives of the study. Therefore, various remote sensing satellites (Table 1) and airborne systems (Table 2) commonly used in water quality assessments, along with their spectral properties including spatial resolution, spectral bands, and revisit interval are presented. This tabulated information is helpful when designing water quality assessment studies, and can be used for the selection of appropriate sensors among many other available sensors in the market.

Other categories of sensors that have broad applications in oceanographic remote-sensing are microwave radiometers (MWR) and synthetic aperture radar (SAR). Passive microwave radiometers measure energy emitted at sub-millimeter-to-centimeter wavelengths (at frequencies of 1–1000 GHz) known as microwaves. By understanding the physical processes associated with energy emission at these wavelengths, oceanographers can calculate two important water quality parameters, sea surface temperature (SST) and sea surface salinity (SSS). They have also been used for measuring atmospheric and terrestrial radiation, meteorological studies, such as zenith-pointing surface instruments that view the Earth's atmosphere in a region above the stationary instrument. Table 3 shows the characteristics of the more commonly used microwave radiometers in oceanography and water quality studies.

Synthetic aperture radar is a form of radar that used to create two or three-dimensional images of objects [32,33,34], and can be mounted on either an aircraft or spacecraft. Although SARs are widely used for water pollution detection like oil pollution, ocean topography, wind speed at the sea surface, and regional ice monitoring, they are not very often applied in water quality studies and measuring water quality parameters. In cases where the data from these sensors are combined with other sensors in water quality studies, the results have demonstrated that SARs are only marginally helpful in improving the estimation of water quality parameters; for example in Zhang [35] study.

## 4. Water Quality Investigations through Remote Sensing Techniques

Water quality study is the process of determining the chemical, physical and biological characteristics of waterbodies and identifying the possible contamination sources that degrade the quality of water [20]. Degradation of the quality of water resources may result from waste discharges, pesticides, heavy metals, nutrients, microorganisms, and sediments. Different water quality standards have been developed to aid in checking the extent of water pollution, and consequently to maintain these quality standards. The most commonly measured qualitative parameters of water are detailed in Table 4.

The terminology and rationale for Case 1 and Case 2 water classifications were established by Morel and Prieur [16] and Gordon and Morel [83] in their seminal work on the bio-optical basis for ocean color variations. The definition for Case 1 and Case 2 waters was updated by Mobley et al. [84] as follows:

- Case 1 waters are those waters whose optical properties are determined primarily by phytoplankton and related colored dissolved organic matter (CDOM) and detritus degradation products.
- Case 2 waters are everything else, namely waters whose optical properties are significantly influenced by other constituents such as mineral particles, CDOM, or microbubbles, whose concentrations do not covary with the phytoplankton concentration.

Remote sensing techniques make it possible to have spatial and temporal view of surface water quality parameters and more effectively and efficiently monitor the waterbodies, and quantify water quality issues. Most of the studies have focused on optically active variables, such as chlorophyll-*a* (chl-*a*), total suspended solids (TSS), and turbidity. There are several other important water quality variables such as pH, total nitrogen (TN), ammonia nitrogen (NH_3_-N), nitrate nitrogen (NO_3_-N), and dissolved phosphorus (DP), which existing literature omit. The main reason is due to their weak optical characteristics and low signal noise ratio. However, these parameters are an important part of water quality indices and are a challenging aspect of research in the field of water quality assessment using remote sensing, which should stimulate and motivate scientists in further efforts. In continuing, the study precisely surveys the more commonly employed approaches in estimating the concentration of the eleven water quality parameters. These water quality indicators include chlorophyll-*a* (chl-*a*), colored dissolved organic matters (CDOM), Secchi disk depth (SDD), turbidity, total suspended sediments (TSS), water temperature (WT), total phosphorus (TP), sea surface salinity (SSS), dissolved oxygen (DO), biochemical oxygen demand (BOD) and chemical oxygen demand (COD).

### 4.1. Chlorophyll-a

**Figure 1:**
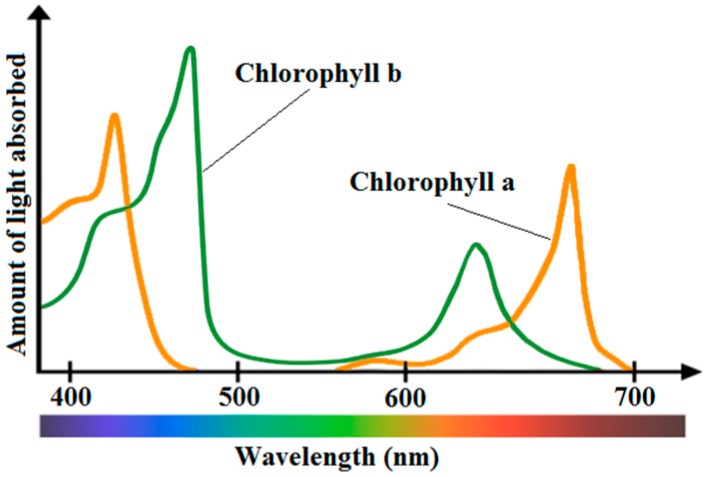
The Absorption Spectrum of both the chlorophyll-*a* and the Chlorophyll-*b* pigments.

Algal blooms, which are often driven by eutrophication phenomena in freshwater, are directly related to chl-*a* concentration since it is essential for photosynthesis [36]. Chl-*a* is used in oxygenic photosynthesis and is found in plants, algae and cyanobacteria. Chl-*a* is the major indicator of trophic state because it acts as a link between nutrient concentration, particularly phosphorus, and algal production. Chl-*a* while mainly reflecting green, absorbs most energy from wavelengths of violet-blue and orange-red light, whose reflectance causes chlorophyll to appear green. Obviously, the addition of chl-*b* besides chl-*a* extends the spectrum absorption. Low light conditions tends to favor the production, a rather greater ratio, of chl-*b* to chl-*a* molecules, thus increasing photosynthetic yield [85]. Figure 1 shows the absorption spectrum of both chl-*a* and chl-*b* pigments. Many researchers have demonstrated that increasing chl-*a* concentration causes a decrease in the spectral response at short wavelengths, particularly in the blue band [86,87,88,89,90,91]. A large number of studies have focused on chl-*a* concentration measurement using remote sensing, some of which are cited in this review paper.

Narrow bands of imagery are required for the measurement of chl-*a* concentration and its spatial and temporal variations within a waterbody [92]. A review of the literature on the application of empirical approaches using multispectral sensors shows ambiguous results. While estimating chlorophyll using remote sensing techniques is possible and a lot of authors claim to achieve satisfying results using broadband sensors, several studies exist showed that the broad wavelength spectral data available on current satellites (i.e., Landsat, SPOT) do not permit discrimination of chlorophyll in waters with high suspended sediments [86]. This is especially due to the dominance of the spectral signal from the suspended sediments, more so in highly turbid and eutrophic waters [93,94].

Since the late 1970’s, many researchers developed bio-optical algorithms that initially were designed for oceans to determine water quality parameters. In these waterbodies, phytoplankton and its breakdown products are the sole determinants of optical properties of the water. In Case 1 waters, by employing an empirical model and interpreting the received radiance at different wavelengths, the concentrations of chl-*a* can be adequately estimated with satellite images [83]. In Case 1 waters, spectral bands in the blue to green region are appropriate to identify chl-*a* concentrations with acceptable precisions. However, in Case 2 waters due to the complexity of the constituents in water, the detection of chl-*a* is a sophisticated task and requires advanced approaches and techniques. In Case 2 waters (inland and coastal waters), the optical properties are determined, additionally to phytoplankton, by a composite of dissolved organic matter from a terrestrial origin, dead particulate organic matter and inorganic particulate matter. Therefore, determination of chl-*a* concentration is much more complex and less accurate as these constituents are not statistically correlated. Additionally, the simple fact that Gelbstoff absorption often masks the blue-green region in Case 2 waters implies chl-*a* algorithms developed for Case 1 waters are not applicable to Case 2 waters [95].

Various visual spectral bands and their ratios are widely used to quantify chl-*a*. Spectral band ratios can reduce irradiance, atmospheric and air-water surface influences in the remotely sensed signal [86,96]. The prominent scattering-absorption features of chl-*a* include strong absorption between 450–475 nm (blue) and at 670 nm (red), and reflectance reaches to peak at 550 nm (green) and near 700 nm (NIR). The reflectance peak near 700 nm and its ratio to the reflectance at 670 nm have been used to develop a variety of algorithms to retrieve chl-*a* in turbid waters [23]. Gitelson [97] studied the behavior of the reflectance peak near 700 nm and concluded that the 700 nm reflectance peak was important for the remote sensing of inland and coastal waters, especially for measuring chlorophyll concentration. Han [98] pointed out that the spectral regions at 630–645 nm, 660–670 nm, 680–687 nm and 700–735 nm were found to be potential regions where the first derivatives can be used to estimate chlorophyll concentration. Dekker et al. [99] mentioned that the scattering and absorption characteristics of chl-*a* can be studied when more than one band is used. Hoogenboom et al. [100] noted that a ratio using an Advanced Visible–Infrared Imaging Spectrometer (AVIRIS) band located near 713 nm along with the band at 667 nm was the most sensitive for chlorophyll retrieval in inland waters. A similar ratio (R674/R705) has been demonstrated to be optimal for inland lakes and rivers [101]. Table 5 shows some of the more commonly used techniques for the measurement of chl-*a* concentration.

Other significant literature that applied other approaches to the measurement of chl-*a* are considered hereafter. Alparslan, Coskun and Alganci [61] measured the concentration of chl-*a* using all bands of Landsat-5 TM. Ekercin [128] used Band 1 (445–530 nm), Band 2 (520–610 nm), Band 3 (640–720 nm), and Band 4 (770–880 nm) of IKONOS data to estimate chl-*a* concentration in Istanbul, Turkey. Also, Nas, Karabork, Ekercin and Berktay [67] used the visible near-infrared (VNIR) and the shortwave infrared (SWIR) (first four bands 0.52–1.70 µm) of Terra/ASTER and developed a multiple regression between chl-*a* concentration and spectral reflectance in the Beysehir Lake, Turkey. Shafique, Fulk, Autrey and Flotemersch [29], using Compact Airborne Spectrographic Imager (CASI) studied the chl-*a* concentration in the Great Miami River and 80 miles of the Ohio River. They concluded that linear models using the ratio of wavelengths 705/675 nm could describe chl-*a* concentration. Bhatti, Rundquist, Schalles and Ramirez [40], using Airborne Imaging Spectroradiometer for Applications (AISA) sensor in the Apalachicola Bay in Florida, USA, found that two bands reflectance ratio R70 Other significant literature that applied other approaches to the measurement of chl-*a* are considered in following. Alparslan, Coskun and Alganci [61] measured the concentration of chl-*a* using all bands of Landsat-5 TM. Ekercin [128] used Band 1 (445–530 nm), Band 2 (520–610 nm), Band 3 (640–720 nm), and Band 4 (770–880 nm) of IKONOS data to estimate chl-*a* concentration in Istanbul, Turkey. Also, Nas, Karabork, Ekercin and Berktay [67] used the visible near-infrared (VNIR) and the shortwave infrared (SWIR) (first four bands 0.52–1.70 µm) of Terra/ASTER and developed a multiple regression between chl-*a* concentration and spectral reflectance in the Beysehir Lake, Turkey. Shafique, Fulk, Autrey and Flotemersch [29], using Compact Airborne Spectrographic Imager (CASI) studied the chl-*a* concentration in the Great Miami River and 80 miles of the Ohio River. They concluded that linear models using the ratio of wavelengths 705/675 nm can describe chl-*a* concentration. Bhatti, Rundquist, Schalles and Ramirez [40], using Airborne Imaging Spectroradiometer for Applications (AISA) sensor in the Apalachicola Bay in Florida, USA, found a significant correlation between the two bands reflectance ratio R700/R670 and chl-*a* concentration. Also, the three band model R750 * (R670^−^^1^ − R700^−^^1^) was found to be a predictor of chl-*a* concentration in Case 2 waters. In addition, the logarithmic ratio of ALOS/AVNIR-2 (Band 3/Band 1) was related with chl-*a* concentration in his study area. Lim and Choi [36] using Landsat-8/OLI showed that chl-*a* presented a good correlation with both OLI bands and band ratio, with calculated R values for Bands 2, 3, 4 and band ratio (Band 5/Band 3) as −0.66, −0.70, −0.64, and −0.64, respectively, at a significance level of *p* < 0.01. ZHANG and HAN [129] found that OLI bands 1 to 4 and their combinations had good correlation with chl-*a* concentration. Kim et al. [130] using Landsat-8/OLI employed Band 2, Band 5, and a ratio of Band 2/Band 4 to measure chl-*a* concentration. Mannheim et al. [131] found that the reflectance curve and the baseline from 672 to 742 nm (CHRIS spectral Bands 8–12) shows the best correlation results and the maximal sensitivity to variations of chl-*a* concentration. Choe et al. [132] used Moderate Resolution Imaging Spectroradiometer (MODIS), Sea-viewing Wide Field-of-view Sensor (SeaWiFS), Medium Resolution Imaging Spectrometer (MERIS), and RapidEye for the estimation of chl-*a* concentration in turbid waters using Two-band and Three-band models based on band ratio such as Red and NIR band.

Furthermore, Qi et al. [133] developed an approach based on Empirical Orthogonal Function (EOF) analysis to estimate chl-*a* concentration in surface waters of Taihu Lake, the third largest freshwater lake in China. The EOF approach analyzed the spectral variance of normalized Rayleigh-corrected reflectance (Rrc) data at 469, 555, 645, and 859 nm, and subsequently related that variance to chl-*a* using 28 concurrent MODIS and field measurements. Feng et al. [134] developed a new empirical chl-*a* algorithm for the largest freshwater lake of China (Poyang Lake) using a normalized green-red difference index (NGRDI) and atmospherically-corrected Medium Resolution Imaging Spectrometer (MERIS) data.

**Figure 2:**
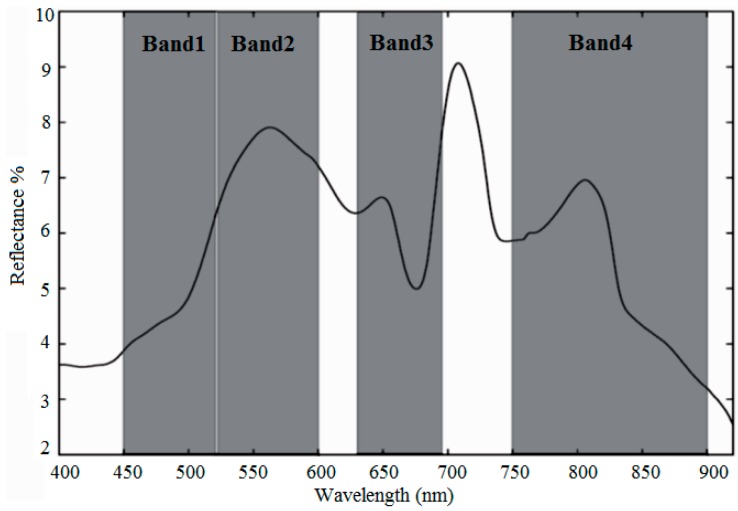
Spectral band positioning of Landsat7/ETM+ on ASD spectroradiometer spectrum [95].

Reviewing the literature showed that most algorithms to determine the chl-*a* concentration need a wavelength near 675 nm and another near 700 nm. For example, the positioning of the spectral bands of Landsat/ETM+ is illustrated in Figure 2. This figure reveals that the bands are not very broad and also not suitably positioned for the detection of chl-*a*. Other multispectral sensors such as ASTER, IRS-LISS III and SPOT-HRV have similar spectral positioning in the Red/NIR region and therefore one can conclude that neither of these sensors is very appropriate for the detection of chl-*a*.

As mentioned, several satellite and airborne imageries can be used for chl-*a* estimation. Nonetheless, it revealed that the Landsat TM seems to be more appropriate and widely used for chl-*a* assessment. Temporal coverage and spatial resolution of TM and its easy accessibility can be the main reasons for the selection of this sensor.

### 4.2. Colored Dissolved Organic Matters (CDOM)

Colored Dissolved Organic Matters, also called gelbstoff and gilvin, consists of naturally occurring, water-soluble, biogenic, heterogeneous organic substances that are yellow to brown in color [135], which exist in both fresh and saline waters. These compounds are brown and can color the water yellowish brown in high concentrations. Therefore, they are referred to as yellow matter or colored dissolved organic matter (CDOM), and usually with chl-*a* and TSS dominate the water color.

CDOM absorbance spectrum can be several times and overlaps the chlorophyll absorption and can account for over 50% of the total absorption at 443 nm, which is the wavelength that chlorophyll concentrations are usually measured [136]. The increase in the CDOM concentration mainly affects the reflectance values in the blue and green region of the spectrum (especially below ~500 nm) and its absorbance increases exponentially with decreasing wavelength. This effect can complicate the use of chl-*a* retrieval algorithms and phytoplankton production models that are based on remotely sensed ocean color [137]. Nonetheless, it is reported by Strömbeck and Pierson [138] that at high CDOM concentrations, absorbance of red light spectrum can be significant.

Remote sensing of CDOM is important in studying aquatic ecology and carbon dynamics [18,139]. Existence of CDOM in rivers, lakes, and oceans affects the water color as seen by many satellite remote sensing instruments, such as MODIS and SeaWiFS [140]. CDOM also affects the underwater light field and water’s inherent optical properties (IOP). This characteristic determines the water reflectance received by remote sensors. Therefore, inversion of remote sensing data provides an efficient method to estimate CDOM concentration within a large spatial and temporal scale [141,142]. In ocean color studies, CDOM absorption properties, for example, its absorption coefficients at 440 nm, are usually used as a representative of CDOM concentration [143]. In the algorithms derived from sensors like CZCS, chl-*a* concentrations were empirically inverted, and accordingly, CDOM can be measured with the assumption that it co-varies with chlorophyll [16,83,144]. Hyperspectral measurements with newly developed remote sensing reflectance models [145] have also been used to estimate CDOM as one of ocean color components, such as EO-1 Hyperion with MIM (Matrix Inversion Method) [8]. Kutser et al. [146] also used band ratio of EO-1/ALI Band 2 and Band 3 to estimate CDOM content in lakes of Southern Finland.

Recent approaches of CDOM estimation combine hyperspectral remote sensing data with semi-analytical models, new factors like the bottom effects, and computational techniques to enhance the accuracy of CDOM inversion [49]. Traditionally, in most water quality monitoring programs, CDOM absorption is referred to as color and PCU color (Platinum-Cobalt Units) and is used to characterize it. In recent studies, CDOM absorption is directly reported as light absorption coefficients at given wavelengths, and these absorption coefficients and PCU colors are closely correlated [136]. Semi-analytical models have been developed and applied to SeaWiFS (Sea-viewing Wide Field-of-view Sensor) and MODIS (Moderate Resolution Imaging Spectroradiometer), in which CDOM’s absorption coefficients are directly and independently inverted from remote sensing reflectance (Rrs) [49]. The semi-analytical models are based on the radiative transfer equations as well as the simplification of radiance and underwater light field [142]. Remote sensing of CDOM in riverine waters and coastal waters is a challenge and subject to large errors compared to oceanic waters because spectral signals of CDOM usually interfere with chlorophyll and suspended sediments [54,147]. In these complex environments, hyperspectral remote sensing present an advantage due to their spectral responses to water inherent optical properties (IOP) and their broad spectrum of narrow bands.

Several studies have confirmed that high spectral resolution (10 nm or better) can improve the estimation of water inherent optical properties (IOP) in coastal water [8,148,149]. However, as mentioned, due to the spectral signal interference from chlorophyll, suspended sediments as well as spatial and temporal heterogeneity of riverine and coastal waters, the applicable bands for CDOM measurement are not always at the same wavelengths. Therefore, identification of significant wavelengths out of hundreds of narrow bands of hyperspectral reflectance is a challenging task [55]. As a solution, first, the dimensionality of hyperspectral data should be reduced through techniques such as band selection, derivative analysis, spectral indices, or hyperspectral transformation [122,150,151,152]. It is also necessary to calibrate and validate the remotely sensed CDOM concentrations using a shipboard data acquisition approach concurrently with high spatial resolution underwater CDOM observation. Additionally, CDOM is reported to be commonly used as an important indicator for dissolved organic carbon (DOC) dynamics in freshwater and coastal marine ecosystems [153] and many observations have provided evidence that CDOM is correlated to DOC [54,154,155,156,157,158]. Reviewing the literature revealed that most of the studies are based on four sections: underwater CDOM measurements, in situ hyperspectral measurements, water-surface reflected radiance by means of remote sensor on a satellite or an airborne platform, and functional data analysis [49,153]. The literature showed that CDOM could be quantified using visual spectral bands and their ratios, which is as summarized in Table 6.

Furthermore, Taheri Shahraiyni et al. [174] by using reflectance values at 490, 510, 560, 620, and 885 nm of MERIS data and applying a fuzzy modeling technique, Active Learning Method (ALM), mapped the spatial distribution of CDOM over the southern parts of the Caspian Sea, Iran. A proxy algorithm was reported for remote sensing of CDOM by an absorption coefficient of ocean water, which is a multi-band quasi-analytical algorithm (QAA) developed by Lee, Carder and Arnone [141]. Further, alternative algorithms such as computer-based discrete modelling methods are developed for remote sensing of CDOM. However, Kishino et al. [175] expressed that results can be questionable when a neural network model is implemented to measure the CDOM concentration using ASTER data. Johannessen et al. [176] using SeaWiFS images found out a relationship between ultraviolet (UV) attenuation coefficient (Kd) at 323 nm, 338 nm, and 380 nm and the Rrs(412)/Rrs(555) band ratio.

Researchers use many sensors to assess CDOM, but SeaWIFS and MODIS, because of their coarse spatial resolution, were widely applied in deep waters. Due to the need for high accuracy for large-scale applications, SeaWiFS data are of little use in shallow waters and hyperspectral imagery like EO-1/Hyperion, EO-1/ALI, and ALOS/AVNIR-2 were preferable for these areas. In addition, a majority of researchers have used a high-resolution spectroradiometer in their in situ hyperspectral measurements to validate their quantified results. These in situ measurements include reflectance values collected at a single certain location and often used as an indicative of individual targets. These data are useful in identifying concentrations of components within the water column and can be collected above and below the water surface [22]. They are also useful for calibration and validation of remotely sensed estimations of water quality parameters.

### 4.3. Secchi Disk Depth

**Figure 3:**
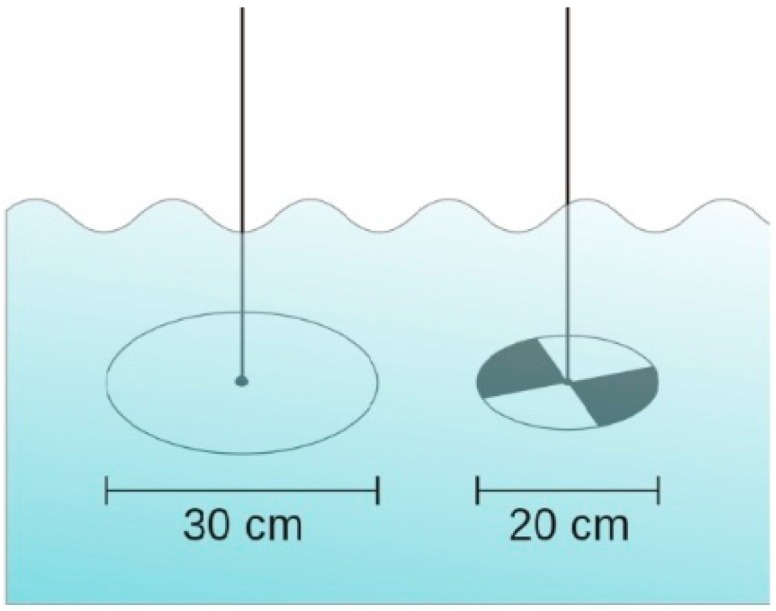
Two different kinds of Secchi disks [183].

Secchi depth is an optical property of water strongly related to water constituents present in the waterbodies. The Secchi depth exhibits an inverse correlation with the amount of total suspended solids (TSS) present in the waterbodies. It can be used to study the relative nutrient and solids loading situations [177]. The most commonly attempted method for the measurement of water transparency is based on light attenuation principles [142]. The best-known operational estimation of water transparency is the Secchi disk, created by Pietro Angelo Secchi SJ in 1865, and is a circular disk used for clarity measurements in oceans and lakes. The disc mounts on a line and lowers slowly down in the water until the pattern on the disk is no longer visible. This measure is known as the Secchi disk depth (SDD) and is also related to water turbidity. Figure 3 shows two different kinds of Secchi disks.

SDD is a reasonable indicator of trophic conditions (algal abundance) except in highly colored lakes with low chl-*a* and non-algal turbidity (clay, calcium carbonate) [42]. The Secchi depth is inversely correlated with the amount of TSS present in the waterbodies. Therefore, remote sensing can be an ideal tool for monitoring water transparency and estimating the SDD. Recently, Lee et al. [178] introduced a model to estimate the SDD, which unlike the classical model that relies strongly on the beam attenuation coefficient, the new model relies only on the diffuse attenuation coefficient at a wavelength corresponding to the maximum transparency for such interpretations. Many researchers have applied remote sensing for this purpose and have shown in their studies that remote sensing data is well correlated with SDD values [179,180,181,182].

SDD has a significant correlation with atmospherically corrected satellite radiance [184,185,186]. In atmospherically corrected MSS Green band, SDD is related to reflectance just below the surface, incorporating the ratio of backscattering to total scattering coefficients for suspended particles [187]. The relationship is quite accurate for SDD < 16 m [188]. Significant algorithms have been developed for SDD using various remote sensing data, like TM [27,39,102,189,190], MSS [24,25,191,192,193], IKONOS [28,60,128] and even video data [194]. Landsat-TM is one of the most frequently used sensors to estimate SDD. Braga et al. [195] found that SDD was closely correlated with TM data, especially during high tide. Furthermore, highly suitable models were developed for SDD that ranged from 4 to 15 m from TM1 and TM3 satellite radiance [186]. However, there was an exception research conducted by Lopez-Garcia and Caselles [124]. They used TM data and reported that SDD did not show significant correlation with any TM bands. SDD can also be quantified from reflected radiance received by the IRS satellite [184].

There are many established relationships in the literature between Secchi depth and total phosphorus, chl-*a*, TSS, and CDOM. The existing literature showed that SDD can be quantified using visual spectral bands and various band ratios. Bhatti, Rundquist, Schalles and Ramirez [40] used ALOS-AVNIR-2 data and found that the Secchi depth was well correlated with reflectance ratio of R750/R560 (NIR/Green). Thiemann and Kaufmann [101] used HyMap and CASI data for Secchi disk transparency and chlorophyll-*a* determination in the Mecklenburg Lake District, Germany. They used the area between a base line and the spectrum from 400 to 750 nm and found a good correlation with the in situ measured Secchi disk transparency (SDT). Ekercin [128] using Band 1 (445–530 nm), Band 2 (520–610 nm), and Band 3 (640–720 nm) of IKONOS data and developed an algorithm for SDD measurements. Mancino, Nolè, Urbano, Amato and Ferrara [26] developed an equation using TM1 and the TM3/TM2, TM1/TM2, TM2/TM1 ratios, and Powell et al. [196] suggested a regression equation related to in-situ Secchi disk transparency measurements by using the Blue, Green, and Red bands of TM. In addition, based on Kloiber et al. (2002) study and TM and MSS imagery analysis, some recommendations were made for a Landsat-based procedure of water clarity assessment. Literature also showed that Secchi disk depth can be quantified using visual spectral bands and various band ratios, which are summarized in Table 7.

Although several satellite remote sensing systems have been used to measure the SDD, the relatively low cost, temporal coverage, spatial resolution, and data availability of the Landsat system make it particularly most useful data for the assessment of this water quality parameter. Several studies have demonstrated a strong relationship between Landsat Multispectral Scanner (MSS) or Thematic Mapper (TM) data and ground observations of Secchi depth, which are cited earlier.

SDD and chl-*a* concentrations have been successfully predicted from satellite image data by developing the relationship between in-situ measurements of SDD and chl-*a*, and the spectral response of the blue, green, red, and near-infrared bands. This approach has been successfully implemented in Minnesota [202], Wisconsin [203], and Michigan [204] to estimate water clarity for inland lakes, where in-situ data is limited.

### 4.4. Turbidity and Total Suspended Sediments

Water turbidity is an optical property of water, which scatters and absorbs the light rather than transmit it in straight lines. Suspended sediments are responsible for most of the scattering, whereas the absorption is controlled by chl-*a* and colored dissolved or particulate matter [205]. As water turbidity is mainly the result of the presence of suspended matter, turbidity measurement has often been used to calculate fluvial suspended sediment concentrations [206] and is commonly regarded as the opposite of clarity. The level of turbidity or murkiness is entirely dependent on the amount of suspended particles in a sample of water. The more suspended particles, the more difficult for light to travel through the water and therefore, the higher the water’s turbidity. The complex nature of suspended substances in water changes the reflectance of the waterbody and therefore causes variation in color. To this end, interpretation of remotely sensed data just based on the color of water is not adequate and accurate. Turbidity and total suspended matters are considered as important variables in many studies due to their linkage with incoming sunlight that in turn affects photosynthesis for growth of algae and plankton. These parameters are also directly associated with Secchi disk depth.

Remote sensing techniques are widely used to estimate and map the turbidity and concentrations of suspended particles, and to provide their spatial and temporal variations. Theory shows that use of a single band provides a robust and TSM-sensitive algorithm provided the band is chosen appropriately [207]. Curran et al. [208] and Novo et al. [209] showed that single band algorithms may be adopted where TSM increases with increasing reflectance. However, the complex substances in water change the reflectance of the water body and therefore cause variation in colors, and thus, different spectral bands can be used for TSS retrievals [207,210,211]. For example, high levels of total suspended solids or the presence of dark-colored humus acids from the decay of vegetation, common in the water of peat bogs, would result in high TSS and turbidity readings (Mark and Stapp, 2003). Therefore, the advantage of using signal band or band ratios can be employed to obtain more accurate results in different concentrations in waterbodies. In the Near-IR and Mid-IR regions, water increasingly absorbs the light and makes it look darker, which varies based on water depth and wavelength. An increase of dissolved inorganic materials in waterbodies causes the peak of visible reflectance to shift from the green region (clearer water) toward the red region of the spectrum. Several studies have also found that the first four bands of Landsat are well correlated with total suspended matters [42,198,212,213]. However, Ritchie et al. [214] by in situ studies showed that the most useful range of spectrum for the determination of suspended particles in surface waters was between 700 and 800 nm. The literature showed that turbidity and/or Suspended Sediments can be measured using visual spectral bands and various band ratios, which are as summarized in Table 8.

Furthermore, Ekercin [128] used Band 1 (445–530 nm), Band 2 (520–610 nm), Band 3 (640–720 nm), and Band 4 (770–880 nm) of IKONOS data and estimated the concentration of TSS in Istanbul, Turkey. Alparslan, Coskun and Alganci [61] obtained the amount of turbidity from Band 1, Band 2, Band 3, Band 4, Band 5 and Band 7 of Landsat-5 TM Satellite Image. He, Chen, Liu and Chen [73] used a combination of Landsat TM Bands 2, 3, 6 and 7 to correlate with the in situ turbidity measurements. Also, Sudheer, Chaubey and Garg [58] suggested that a combination of TM1, TM2, TM3 and TM4 was significant to retrieve suspended sediments information from remote sensing data. Bhatti, Rundquist, Schalles and Ramirez [40] by using NIR/Green band ratio of ALOS-AVNIR-2 developed a relationship to calculate total suspended matters. Lim and Choi [36] found that suspended solids was correlated with Bands 2–5 of Landsat-8/OLI, and constructed 3 multiple regression models through single bands of OLI.

Reviewing the literature demonstrated that the Landsat/TM was used much more than other sensors. For rivers and other case studies that need more spectral and spatial resolution, ALOS/AVNIR-2, IKONOS on spaceborne sensors, and CASI and AISA hyperspectral imagery on airborne sensors were used to determine turbidity and suspended matters. The methodology to interpret images and to evaluate the turbidity was also improved from simple linear regression to non-linear multiple regression, principle components analysis (PCA) and neural networks.

### 4.5. Total Phosphorus

Total phosphorus (TP) studies consist of the measurement of all inorganic, organic and dissolved forms of phosphorus. Phosphates are plant nutrients whose increased quantity helps plants and algae to grow quickly. Total phosphorus can be directly related to chl-*a* concentration and indirectly related to transparency or water clarity, which is estimated by Secchi depth [218]. Rivers that flow through various land use activities can include different substances and chemicals like total suspended sediments, nutrients, residential fallout, and others. When a river or a creek passes through an agricultural area, for instance, the phosphorus load may show a higher concentration compared to other parameters present in the surface water. Fertilizer-rich agricultural runoffs and effluents from wastewater treatment plants are the main sources of high phosphorus and nitrogen concentrations in surface waters that threaten many worldwide ecosystems [219]. Total suspended matters usually act as a carrier for TP and also closely related to Secchi disk transparency with an exponential equation [220].

The measurement of total phosphorus concentrations in waterbodies is challenging due to the spatial heterogeneity and the labor-intensive collection and testing of required field samples. Remote sensing as a robust tool has already been used successfully to monitor water quality parameters in various scales and areas, although it presents a challenge in estimating phosphorus concentration. Remote estimation of total phosphorus (TP) has been investigated based on its high correlation with optically active constituents [69,220,221,222,223,224]. Total phosphorus is not directly measurable by optical instruments, but has a general correlation with other water quality parameters. As mentioned above, TP is closely related to some other parameters like phytoplankton [220,223], turbidity and total suspended matters (TSM), and Secchi disk transparency (SDT) [224], which is the basis for remote monitoring of TP dynamics [225]. Multispectral Landsat TM data have been widely used to monitor and map the TP spatial and temporal pattern in different regions [69,221,222]. Hyperspectral airborne or spaceborne remote sensing due to its finer diagnostic spectral band(s) provides more potential to detect TP in rivers and small lakes.

Many studies have shown that increasing the TP concentration in waterbodies results in a general tendency of increase in chl-*a* concentration [226,227,228,229]. Schindler [230] showed that 74% of the variability in chl-*a* concentration among lakes has a direct correlation with the variation of phosphorus concentration. His result suggests that chl-*a* concentration may play a role as a proxy of phosphorus concentration in waterbodies. In another study conducted by Heiskary and Wilson [231], the Secchi disk depth was decreased with increasing TP concentration that proved that a proportion of phosphorus can be attached to suspended particles resulted from soil erosion and transferred through river’s downslope. These studies suggested that both chl-*a* concentration and SDD are closely correlated with TP concentration [220] and therefore can be used as the potential theoretical parameters for the indirect prediction of TP concentration.

Table 9 shows a number of investigations to measure total phosphorus by applying blue band (0.45–0.51 μm) and green band (0.50–0.60 μm), and integration of red (0.60–0.70 μm) and green (0.50–0.60 μm) ratio from different sensors. Empirical estimations and various statistical regression models were used to correlate phosphorus concentration with other water quality indicators, such as Secchi depth (SD) and chl-*a* concentration. In addition, Bistani [232] using EO-1/Hyperion obtained a reflectance determination coefficient of 0.49 from the 467 to 529 nm bands ratio values, from which he derived a polynomial algorithm used to produce a total phosphorus distribution map. Song et al. [233] studied the correlation between TP and TM1, TM2, TM3, and TM4 from the Landsat 5, and found that each band had a correlation with TP of 0.62, 0.59, 0.55, and 0.51, respectively. Later in another study, Song, Li, Li, Tedesco, Hall and Li [68] by using the airborne imaging data (AISA), and applying red band (around 690 μm) and NIR spectral region (around 710 μm) estimated the total phosphorus (TP) in three central Indiana water supply reservoirs. Wu, Wu, Qi, Zhang, Huang, Lou and Chen [69] used a combination of TM1, TM3/TM2, and TM1/TM3 data to correlate chl-*a* concentration and SD measurements with TP concentration. Also, Alparslan, Coskun and Alganci [61] using Band 1, Band 2, Band 3, Band 4, Band 5 and Band 7 of Landsat-5 TM Satellite Image obtained the amount of total phosphorus concentration. Lim and Choi [36] used Bands 2, 3, 4, and 5 of Landsat-8/OLI, and constructed 3 multiple regression models by selecting both single bands and band ratios, and obtained significant correlation coefficients.

Results from studied articles indicate that there is a potential to estimate total phosphorus concentration at different scales using airborne and satellite images. The Landsat/TM was used much more than other sensors for TP assessment in the reviewed literature. As phosphorus does not directly present optically diagnostic signals in water leaving radiance for the water quality remote sensing spectral domain (400–900 nm), thus empirical modeling is considered the most applicable approach for the remote estimation of TP in water column [68,69,235]. The literature review also showed that TP has a similar spatial pattern to chl-*a* and SD concentration due to a high correlation of TP with these parameters. Total phosphorus also was highly related to sediment loadings. However, there is a time lag for phytoplankton to consume TP in reservoirs, which make the relationship between TP and chl-*a* or SD and total suspended sediments more complicated [68].

Light reflection from the bottom in shallow waters cannot be very reliable, because it may be a result of the above-water remotely sensed reflectance spectra. Therefore, the TP concentration estimated in shallow water may be questionable and needs to be validated using in situ data. Spatial and temporal distribution algorithms for TP concentration produced from satellite-based observations should also be verified by in situ measurements. These empirical methods provide site-specific predictions of total phosphorus with reasonable accuracy [236].

### 4.6. Water Temperature

Water temperature is an important parameter for the physical and biochemical processes occurring within water as well as in air-water interactions because temperature regulates physical, chemical, and biological processes in water. Water temperature also influences the solubility, and thus availability of various chemical constituents in water. Most importantly, this parameter affects dissolved oxygen concentrations in water; as oxygen solubility decreases with increasing water temperature. It is also very important to analyze the temporal variations due to seasonal changes. On the other hand, distribution, transportation, and interaction of some contaminants, such as nutrients have a significant relation with water column temperature.

Thermal infrared bands are able to measure the amount of infrared radiant heat emitted from land surfaces and the radiant temperature of waterbodies that have environmental and economic import. As thermal infrared is emitted from the surface, temperature estimations derived by remote sensing must be evaluated with great care when there are reasons to assume that the water is stratified [237], which occurs shortly after precipitation or solar warming and in areas influenced by freshwater runoff. In such cases, no relation can be expected between sea surface temperatures and the temperatures found in the water under the surface. Thermal stratification in freely flowing rivers is inherently unstable due to variations in channel shape, and in-stream objects, which cause a turbulent flow regime and can usually be detected in the imagery [238].

Remote sensing of water temperature in rivers is more complex than in other waterbodies because of their much smaller dimensions and difficulties of determination at the resolution (pixel size) of the thermal-infrared (TIR) data [45]. Stream and river temperature is crucial especially when dealing with endangered fish populations, which are sensitive to increased water temperature. Sparse sampling in both space and time restricts traditional assessment of water temperature, which is typically measured using a network of in-stream gauges, and records the temporal change at given locations. These gages, located in main streams and rivers, are limited in terms of spatial distribution of river temperatures. The application of remote sensing techniques can be an attractive alternative to measuring and monitoring stream temperatures with determined accuracies and uncertainties. Remotely sensed TIR images could provide reliable measurements of the spatial distribution of the stream and river temperature. Remote measurements of water temperature can be obtained with a sensor that detects thermal radiation (3–5 and 8–14 μm wavebands) emitted from the upper 0.1 mm of the water surface [239,240,241,242].

The emitted TIR radiation (3–14 μm) is a well-established practice, particularly in oceanography where daily observations of regional and global sea-surface temperature (SST) are made from satellites [7,243,244,245]. In the terrestrial environment, TIR remote sensing of surface water temperature initially focused on lakes [246,247], and coastal applications such as thermal pollution from cooling water discharge from a power plant [248]. However, starting in the 1990s, airborne TIR remote sensing has been conducted by government agencies over thousands of kilometers of rivers to monitor water quality, identify sources of cold-water inputs, and to develop spatially referenced river temperature models [249,250,251]. Currently, many TIR imaging sensors are available that have multiple spectral bands located at different wavelengths, which make them suitable for water temperature measurements. For the selection of appropriate band or bands, careful consideration on the least amount of instrument noise and atmospheric effects is necessary for accurate calculation of the water temperature. However, multiple bands can be averaged to reduce noise due to atmospheric or sensors differences and provide a better estimate of the actual temperature [45].

Spaceborne TIR imaging sensors cover greater aerial extents compared to airborne TIR imaging sensors. However, significant differences in their range of pixel sizes, number of bands, revisit times, and sensor sensitivities exist. TIR satellite images are an attractive source of broad-scale data due to their low cost, capability for regional coverage, and revisit times, if they are available for the study time, and also have a suitable pixel size. Airborne sensors with finer pixel size are necessary for smaller waterbodies like rivers, but these images are limited to use over large areas because of the high expense of calibrating and processing. In riverine environment, airborne TIR imaging sensors are widely used for monitoring water temperature. When using airborne data acquisition, it is imperative to consider that these images do not provide a truly synoptic assessment of water temperature at a particular time, if the images are collected consecutively along the river course. Therefore, diurnal changes in water temperature should be considered in planning airborne data collection [252]. TIR imaging system must also be able to minimize internal drift such that frame-to-frame measurements are consistent. In addition, in the case of frame based TIR imaging sensors, the TIR accounting for radiometric distortion must be considered due to variability in individual detector response and lens optics. These uniformity corrections can be performed internally or during the post processing [253].

Water temperature is a good indicator of the vertical mixing condition and water mass type, and can be used to estimate primary production and phytoplankton growth rates. Preliminary studies have shown that the application of remote sensing combined with traditional in situ temperature measurements can provide reliable information on temperature zones at a relatively low cost. Many studies have shown the applicability of remote sensing to temperature estimation for rivers and streams. For example, Torgersen, Faux, McIntosh, Poage and Norton [251] used fine pixel-size (0.2–0.4 m) airborne TIR images to evaluate the accuracy of radiant temperature measurements, and found that the remotely sensed radiant temperature was within 0.5 °C of in-situ measurements. They identified that reflected TIR radiation, vertical thermal stratification in the stream, and thermal boundary-layer effects at the water surface should receive greater attention in the thermal remote sensing of streams. They also concluded that fine pixel-size measurements of stream temperature are useful for studying fine-scale spatial variation and patterns in stream temperature related to hydrological features such as ground-water inputs.

Accurate remote sensing measurement of sea surface temperature (SST) is also vital for weather and climate operational as well as atmosphere studies. Infrared radiometers yield SST to around 0.5 °C precision, though its use is limited in shady zones due to the presence of clouds or fog. Therefore, standard remote sensing practices should be applied to identify and mask these issues out of the used images before one proceeds with the measurement of the water temperature by TIR radiation. Passive microwave techniques are used in cloudy areas with an accuracy limit of about 1.5–2 °C by the relatively large variation of microwave emissivity with surface conditions, such as wind speed [254]. Addition of active microwave (radar) observations can enhance the precision of passive microwave estimates of SST. Reviewing the literature indicates the use of infrared thermal band for quantifying water temperature, which is as summarized in Table 10.

### 4.7. Sea Surface Salinity (SSS)

Salinity and temperature are important factors to identify the density of seawater, and in turn, density is a critical component driving the currents in the oceans. Therefore, salinity is one of the key variables worth considering when monitoring and modeling the circulation in oceans. The role of ocean circulation in moderating the climate is crucial, and thus, sea surface salinity (SSS) is also critical to determine the global water balance, productivity forecast models, as well as evaporation rates. For example, when the salinity is relatively low, the mixed layer will be more stable, and the nutrient pump may be partially inhibited, possibly leading to reduced productivity or a delay in the onset of spring and autumn phytoplankton blooms [288]. Seasonal and inter-annual variability of sea surface salinity represent limitations on the hydrologic balance and coupled ocean-atmosphere climate models [288,289]. Salinity plays a crucial role in the air-sea exchange of gases.

Precipitation makes the ocean water fresher and less dense, which overlays the salty water below, and this thin layer of sea surface fresh water can spoof the shallow satellite readings. The effect of this phenomenon in the tropical ocean, where heavy rainfalls can create pools of local fresh water, is more sensible. It can increase the stability of the upper layer of the water column and significantly reduce the rates of gas transfer across the pycnocline. Spatial and temporal variations in salinity are greater in inland waters and gulfs because they are strongly influenced by climatic events like precipitation vs. evaporation, seasonal river runoff variations, and exchanges with oceans due to tides and flushing times. Spaceborne and airborne experiments in the 1970s and 80s proved the potential of passive L-band microwave radiometers for the measurement of SSS.

Satellite remote sensors provide more frequent and higher spatial resolution data and also make observations at high latitudes. As the measurement of surface salinity by passive microwave radiometers requires long wavelengths (20–30 cm), accurate estimation of SSS from satellite altitudes would require an enormous antenna, which most satellites could not accommodate [290]. New interferometric technology has made it possible to overcome such problems with antenna size [291,292]. For instance, the Moisture and Ocean Salinity satellite (SMOS) has been in use to measure SSS and provide synthesized SSS maps with a high accuracy. It employs the Microwave Imaging Radiometer using Aperture Synthesis (MIRAS), as the primary instrument on the SMOS, with a fixed two-dimensional interferometric antenna, which operates over a range of incidence angles and makes it different from the old passive microwave imagers. The SMOS operates in a sun-synchronous orbit at an altitude of 760 km with a three-day repeat cycle [291,292,293].

Aquarius is another salinity-related sensor that provides the global view of salinity variability required for climate studies. Aquarius was launched on June 10, 2011 and was a NASA microwave radiometer aboard the Argentine. Aquarius L-band radiometer and a scatterometer instrument combination was designed to provide global salinity maps on a monthly basis with a spatial resolution between 76 and 156 km, a swath width of 390 km, and an accuracy of 0.2 psu. The Aquarius was mounted on SAC-D (Scientific Application Satellite-D) and was designed to provide high precision SSS data and monitor the annual and seasonal variation of the large-scale features of the surface salinity field in the open ocean. Additionally, it had the capabilities of conducting long-standing studies regarding how the oceans respond to climate change and the water cycle. For instance, changes in freshwater input and output to the ocean, associated with precipitation, evaporation, ice melting, and river runoff, could be obtained from monthly SSS maps. In addition, Aquarius data are useful for tracking the formation and movement of huge water masses that regulate ocean circulation and the Earth’s climate (NASA, 2011). The Aquarius instrument successfully achieved its science objectives and completed its primary three-year mission in November 2014. Airborne microwave radiometers, such as the Scanning Low-Frequency Microwave Radiometer (SLFMR) and the Salinity, Temperature, and Roughness Remote Scanner (STARRS), have also been used successfully to map SSS and its variability in estuaries and coastal waters.

Many researchers have also used indirect methods based on, for example, satellite-derived temperature profiles, brightness temperature, and CDOM to determine SSS variability. As the salinity has no direct color signal, it could be rather estimated the color signal dominated by major water constituents and developed relationships, such as: (I) Relationship between salinity, temperature, and brightness temperature [64,288,294,295,296,297,298,299,300,301]; and (II) Relationship between salinity and CDOM [54,166,302,303]. Remote sensors on aircraft and satellites offer a means for making detailed SSS measurements over large coastal and ocean areas. Some of these experiments are as listed in Table 11, based on the used sensor.

Other notable experiences are performed using European Remote Sensing satellite (ERS) C-band scatterometer [304]; the first seven bands of MODIS [305]; TOPEX/Poseidon Microwave Radiometer [298,306], and Cooperative Airborne Radiometer for Ocean and Land Studies (CAROLS) L-Band Radiometer [64]. Nonetheless, a comparison of the various sensors’ characteristics shows that the airborne ESTAR and SLFMR are more appropriate than other instruments to sea surface salinity measurements. That notwithstanding, SMOS and Aquarius are the most widely used sensors for the remote sensing of salinity.

Salinity-measuring satellites require extensive procedures for internal and external calibration and validation to ensure the quality of the geophysical data. These processes are based on characterization measurements, which are performed initially on the ground before launch and, subsequently, in-flight. The calibration scheme encompasses both the spaceborne instrument and the ground data processing. Once the system has been calibrated, the instrument performance can be verified, and the ocean salinity can be measured with a higher level of confidence [307,308].

### 4.8. Dissolved Oxygen (DO), Biochemical Oxygen Demand (BOD) and Chemical Oxygen Demand (COD)

Dissolved oxygen (DO) is a crucial water quality parameter that influences the living conditions of all aquatic organisms that require oxygen. The level of DO in waterbodies can be affected by anthropogenic activities and natural occurrences in catchments. Water temperature, the amount of oxygen taken out of the system by respiring and decaying organisms, and the amount of oxygen put back into the system by photosynthesizing plants, stream flow, and aeration are the factors that control the amount of dissolved oxygen in waterbodies. The water temperature highly influences the amount of DO; in other words, less oxygen dissolves in warm water than cold water.

Biochemical Oxygen Demand (BOD) is a measure of the amount of oxygen that bacteria will consume under aerobic conditions while decomposing organic matters. Among the sources of food for water-borne bacteria include among others natural organic detritus and organic waste from waste water treatment plants, failing septic systems, as well as agricultural and urban runoff. By exploiting dissolved oxygen, the bacteria decompose these organic materials resulting in a reduction in the level of DO necessary for supporting aquatic life. A simple means of determining the biochemical oxygen demand is by incubating a sealed sample of water for five days and measuring the loss of oxygen from the beginning to the end of the test. Noteworthy is that the need to dilute the samples prior to incubation stem from the likelihood of the bacteria depleting all the oxygen available in the bottle before the test is complete.

Chemical oxygen demand (COD) is the quantity of matter measured with chemical method that needs to be oxidized in water, especially organic contamination. In the context of chemical oxygen demand, no differentiation exists between biologically available and inert organic matter, and COD is largely a measure of the total amount of oxygen required to oxidize all organic material into carbon dioxide and water. BOD values are always less than COD values, yet measuring the latter take only a few hours while measuring BOD takes five days.

Any discharge of effluent with high BOD levels into a stream or river spontaneously accelerates bacterial growth in the river, which in turn consumes and thus reduces the oxygen levels of the water. One pertinent catastrophe is that the oxygen may diminish to levels that are lethal for most fish and many aquatic insects. However, as the river re-aerates due to atmospheric mixing coupled with algal photosynthesis that adds oxygen to the water, the oxygen levels will slowly increase downstream. Routine methods to measure COD are based on points, and have the time-consuming and laborious disadvantages in obtaining the distribution patterns so that it is difficult to reflect the status of whole region synchronously. This kind of point sampling methods may give accurate measurements, but they are time and money consuming. Further and most importantly, they cannot provide the real-time spatial overview that is necessary for the global assessment and monitoring of water quality [90]. Satellite remote sensing may provide suitable ways to integrate aquatic data collected from traditional in situ measurements.

A review of the available literature confirmed that no single identified and/or recommended sensors can be used with high confidence to perform an appropriate model to measure the reflectance of water resulting from DO, COD, and BOD. There are some examined statistical techniques to determine the relationship between the satellites estimated reflectance and physicochemical parameters of water. Several water quality models were developed to investigate the relationship between the measured values of DO, BOD, and COD in laboratory and remote sensing reflectance, by establishing linear, exponential, and logarithmic regressions. Also, various bands ratios have been studied to obtain the DO, BOD, and COD distribution maps in order to analyze the spatial and temporal changes of these water quality parameters. However, interpretation of the satellite or airborne images and making authentic relationships between spectral characteristics of images and in situ measurements of DO, BOD, and COD in the aquatic ecosystems are still poorly understood. The most notable studies to estimate the amounts of DO, BOD, and COD are as cited in Table 12.

Although the results of studied articles indicated that the Landsat/TM was used much more than other sensors to estimate the amount of DO, BOD and COD, this research found relatively low potential and accuracy of current remote sensing techniques for the measurements of DO, BOD and COD values in waterbodies, unless there are enough and adequate ground proofs. In situ measurements of surface water radiation and atmospheric corrections of images are vitally important for both the calibration and validation of remotely sensed data.

Despite the fact that remote sensing can be used to reflect many of water quality parameters, such as Secchi disk depth, chlorophyll concentrations, CDOM, total suspended sediments, and temperature, emphasis should be placed on the fact that this technique cannot substitute the traditional methods. The reason behind this is that some parameters of water quality, like DO, BOD and COD cannot be determined with a high level of confidence by these techniques. However, remote sensing techniques are valuable and important for remote areas where direct access is not easy and where the sum of sampling and chemistry analysis costs is high.

## 5. Limitations of Remote Sensing for the Assessment of Water Quality Parameters

Remote sensing is a suitable technique to study the spatial and temporal variations of water quality variables. However, a number of important constraints that require precise considerations prior to conducting this technique. Developed models from remote sensing data require adequate calibration, and validation using in situ measurements, and can be used only in the absence of clouds. Moreover, the accuracy of extracted water quality parameters might be debatable for some situations; for instance, Kutser [170] contends that the densest areas of cyanobacteria blooms in the Baltic Sea are usually undetected by standard satellite products due to atmospheric correction or processing errors.

The spatial, temporal, and spectral resolution limitations of many current optical sensors can confine the application of remotely sensed data to assess water quality. Furthermore, certain key parameters that are not easy to measure directly by optical sensors exist, examples of which include water discharge and vertical distribution of water quality parameters in waterbodies. The cost of hyperspectral or airborne data, as well as the required equipment for in situ hyperspectral measurement, is among the main restrictions of using remote sensing methods for water quality assessment. The optical complexities of inland and coastal waters also narrow the scope of remote sensing application [40].

The segregation of spectral signatures for chl-*a*, CDOM, and inorganic suspended matter is not well documented in the literature, which is challenging because of the influence of these parameter on each other. The atmospheric interference also restricts the optical signals coming from waterbodies. In the clear sea waters, the maximum light penetration depth expected is about 55 m near 475 nm [330], and the majority of the incident energy on the water surface is absorbed, and/or transmitted. On the other hand, when the concentrations of suspended sediment extend to say 400 mg/liter, the penetration depth reduces to only 60 cm. Therefore, a progressively thinner layer of surface water is detectable [40].

Most of the studies have focused on optically active variables, such as chl-*a*, CDOM, TSS, and turbidity. However, a number of important water quality variables such as PH, total nitrogen (TN), ammonia nitrogen (NH_3_-N), nitrate nitrogen (NO_3_-N), and dissolved phosphorus (DP), etc. are not well investigated due to their weak optical characteristics and low signal noise ratio. Despite the mentioned limitations, remote sensing is still a useful tool for water quality monitoring.

## 6. Discussion

Several satellite images could be used for water quality assessments. Nonetheless, Landsat TM (Thematic Mapper) images have been used extensively due to their relatively low cost, temporal coverage and spatial resolution [3,11,12,26,42,58,61,73,86,88,102,103,105,106,109]. TM data resided on Landsat-5, a sensor that was operational from 1984 until November 2011, and is considered one of the oldest sensors still used for water quality assessment today [39,61,69,213,216,331]. Results from earlier studies referenced in tables indicate that the resolution of Landsat TM is suitable for water quality studies.

Information from the available literature revealed that the Landsat sensors, TM (Thematic Mapper), MSS (Multi-Spectral Scanner), ETM (Enhanced Thematic Mapper), and OLI (Operational Land Imager) have been used fairly successfully to measure most of the important water quality parameters, such as chlorophyll-*a*, Secchi disk depth, Total phosphorus, Total Suspended matters, Turbidity, Dissolved Oxygen, Biochemical Oxygen Demand, and Chemical Oxygen Demand [10,24,39,42,61,75,130,196]. Nonetheless, the use of Landsat data for measuring water quality characteristics has important limitations. The repeat cycle of 16 days imposes major limitations on intra-seasonal monitoring, especially in areas characterized by frequent cloud cover. The water quality parameter characteristics must be related to an “inherent optical property” (IOP) that can be measured by the satellite sensor [42]. For instance, Kloiber, Brezonik and Bauer [24] related Secchi disk transparency (SDT) to the radiance measured by Landsat TM and MSS in several spectral bands. Some other potential sources of error due to varying atmospheric conditions are mentioned for Landsat. For example, Brezonik, Menken and Bauer [42] noted that the measurements of the radiance at the TM sensor are not calibrated for the intensity of incoming solar radiation, which varies with latitude, season, and time of day. Atmospheric interference can be significant over waterbodies because atmospheric haze scatters light (especially blue wavelengths), and the potential for such interference increases as reflected radiance from the water decreases; thus, lake waters with high clarity and high algae and CDOM are most affected. These restrictions may apply to other sensors with similar characteristics to Landsat sensors.

Although, spaceborne hyperspectral sensors with low spatial resolutions, such as MERIS and MODIS have been used for many years to monitor coastal and large inland waterbodies, currently, there are not many hyperspectral sensors in space with suitable spatial resolution capable of monitoring small lakes and ponds. Due to its spatial resolution of 30 m, the hyperspectral imaging spectrometer Hyperion on the EO-1 platform, launched in November 2000, gave rise to new possibilities of operational monitoring [332]. The Compact High Resolution Imaging Spectrometer (CHRIS) on the platform PROBA with a ground resolution of 18 m complies with these requirements as well. However, since it is a technology demonstrator, it is not in operational use.

Spaceborne and airborne remote sensing and their characteristics, advantages, and disadvantages were discussed previously in this paper. Different considerations of a project, such as required spatial and spectral resolution, geographic coverage area, and project budget determine the preference of one sensor or another. Table 13 represents a summarized comparison of the previously discussed issues related to spaceborne and airborne sensors, where various parameters of these sensors are compared.

Morel & Gordon [333] distinguished three different approaches for estimating concentration of water quality parameters; empirical, semi-empirical, and physical or analytical approach. Empirical approaches seek statistical relationships between spectral bands or band combinations and the measured water parameters, without including knowledge of spectral characteristics of the constituents or any physical explanation of the relationship. Semi-empirical methods utilize the physical and spectral information (e.g. absorption features) to develop the algorithms, which are then correlated to the measured constituents. The statistical coefficients are typically bound to the specific region and time of calibration. Analytical approaches determine the constituents’ concentration by modeling the reflectance of surface water and utilizing the inherent and apparent optical characteristics. However, the semi-analytical approaches use simplified analytical models.

The empirical approaches are easy to implement and require less mathematical skills and computation time. Nonetheless, these methods are not suitable for parameters which do not represent distinctive absorption features, like CDOM and to a certain extent for suspended matters [334]. The analytical approaches can simultaneously determine all constituents of water if the inherent properties of the parameters are well known and large amounts of in situ data are accessible.

## 7. Conclusions and Recommendation

By increasing the anthropogenic activities and industrial development, water quality has dramatically degraded. Remote sensing and GIS techniques in conjunction with traditional in-situ sampling are the most effective, cheaper and more reliable tools for monitoring water quality parameters in various waterbodies (lakes, rivers, ground water, etc.). From the available literature, one pertinent deduction is that various space-borne and airborne sensors can measure water quality parameters with reliable precision. Newly developed hyperspectral satellite imageries, which can simultaneously record up to 200 spectral channels, such as the Hyperspectral Imager for the Coastal Ocean (HICO), are much more powerful systems for detecting water quality parameters. Also, hyperspectral airborne sensors have greater potential because of their simultaneous collection of narrower and contiguous bands that allow various parameters of water quality to be measured and monitored. Therefore, monitoring and assessing water quality issues through remotely sensed data can result in effective management of water resources. However, few managerial decisions rely on remote sensing-derived water quality evaluations. Instead, current methods for measuring water quality focus on periodic (boat-based) or continuous (ship-based or buoy-based) monitoring models. To best realize the full application potential of remote sensing technologies, an open and effective dialogue is needed between scientists, policy makers, environmental managers, and stakeholders at federal, state, and local level. Results from an internal US Environmental Protection Agency qualitative survey performed by Schaeffer et al. [335] were used to determine perceptions regarding the use of satellite remote sensing for water quality monitoring to begin understanding why management decisions do not typically rely on satellite-derived water quality products. They also pointed out that difficulties in developing solutions to clarify the perceptions of environmental managers, identified 22 years ago by Specter and Gayle [336], still exist today.

In most cases, managers and policy makers without technical expertise typically lack the knowledge to understand technical descriptions, abilities and limitations of remote sensing techniques. Therefore, it is essential to clarify perceptions of water quality managers, which does not seem to be necessarily simple or readily achievable. It is highly recommended that researchers, who work in the field of optics and remote sensing beyond publishing manuscripts in peer-reviewed journals, continue to communicate more with water resource management agencies on using appropriate available tools to address important monitoring requirements.

As illustrated in this paper, both satellite and airborne remote sensing are useful in assessing the quality of inland waters. Airborne sensors are more flexible tools than spaceborne sensors because of their higher spatial and spectral resolution coupled with their greater number of spectral bands that makes it possible to retrieve the water quality parameters with more accuracy. Airborne sensors are more suitable to monitor smaller waterbodies, such as rivers and their tributaries, ponds, and estuaries, while satellite sensors are more suitable for the observation of larger waterbodies. In this paper, various properties (spectral, spatial and temporal, etc.) of spaceborne and airborne sensors are tabulated to be used as a sensor selection guide in related studies. Furthermore, based on the literature surveyed, this paper compiled a list of sensors that have been used by researchers to measure various water quality parameters, and compares various parameters of spaceborne and airborne sensors.

Due to the need for high accuracy in local-scale and riverine studies, some of the above mentioned sensors, such as SeaWiFS data are of little use. For these cases, the high resolution and/or hyperspectral remote sensing on spaceborne platforms such as EO-1/Hyperion, ALOS AVNOR-2, IKONOS, HICO, and Landsat-8 and airborne platforms CASI, AISA, AVIRIS, HyMap are recommended for use in water quality measurements.

**Figure 4:**
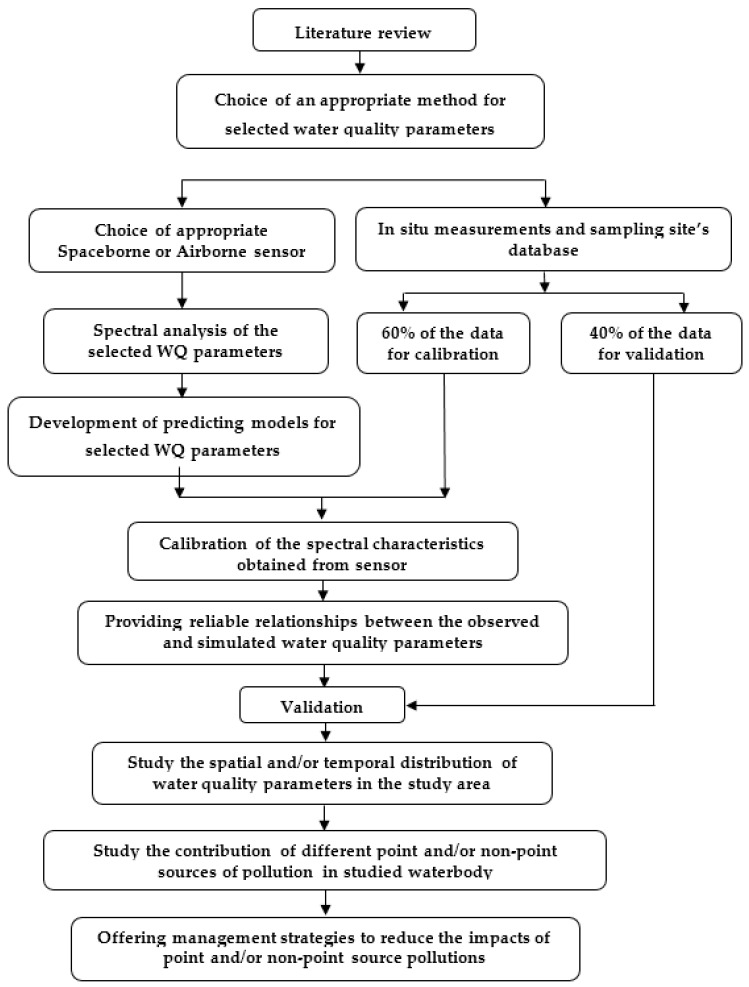
A suggested remote sensing based framework to predict and assessment of water quality variables.

In addition, the recent advances in computer sciences have had a profound influence on the water quality monitoring, resulting in a broader development of the remote sensing technology. Computers can store and analyze the large sets of data generated by most of the Remote Sensing projects. Also, the use of decision support systems (DSS) and Geographical Information Systems (GIS) provide efficient tools for storing, manipulating and analyzing remote sensing data. GIS can enhance the contributions of water quality modelling for practical water quality forecasting, which is essential for sustainable water resources management and development. Therefore, the excellent practicality and interoperability of the RS and GIS techniques will lead the future water quality models towards integration of RS and GIS techniques and the increased use of these technologies in qualitative studies of water resources. Regardless of numerous endeavors reported in the literature, remote sensing techniques utilized to quantify water quality are yet to be adopted on a routine framework. Based on author’s prior knowledge and experience, and the gained information from this literature review, a schematic flowchart of the supposed framework for water quality monitoring and assessment using remote sensing techniques is presented in Figure 4. Despite the recent development of analytical approaches, empirical and semi-empirical algorithms are still in extensive use due to the complexity of analytical approaches in terms of their theory and calculation difficulties. Improvement of the methodology to interpret images from simple linear regression to multivariate statistical analysis approaches like principle components analysis (PCA) and neural networks will help to make the procedures more accurate and easier to manipulate.

## Figures and Tables

**Table 1:** List of the more commonly used spaceborne sensors in water quality assessments.

Category	Satellite—Sensor	Launch Date	Spectral Nands (nm)	Spatial Resolution (m)	Swath Width (km)	Revisit Interval (Day)
**High Resolution**	Digital Globe WorldView-1	18 September 2007	Pan	0.5	17.7	1.7
	Digital Globe WorldView-2	8 October 2009	8 (400–1040)-1 Pan (450–800)	1.85–0.46	16.4	1.1
	NOAA WorldView-3	13 August 2014	8 (400–1040)-1 Pan( 450–800)-8 SWIR (1195–2365)	1.24–3.7–0.31	13.1	1–4.5
	Digital Globe Quickbird	18 October 2001	4 (430–918)-1 Pan (450–900)	2.62–0.65	18	2.5
	GeoEye Geoeye-1	6 September 2010	4 (450–920)-1 Pan (450–800)	1.65–0.41	15.2	<3
	GeoEye IKONOS	24 September 1999	4 (445–853)-1 Pan (526–929)	3.2–0.82	11.3	~3
	SPOT-5 HRG	4 May 2002	3 (500–890)-1 Pan (480–710)-1 SWIR (1580–1750)	2.5 and 5–10–20	60	2–3
	CARTOSAT	5 May 2005	Pan (500–850)	2.5	30	5
	ALOS AVNIR-2	24 January 2006	4 (420–890)-1 Pan (520–770)	2.5–10	70	2
**Moderate Resolution**	Landsat-8 OLI/TIRS	11 February 2013	5 (430–880)-1 Pan (500–680)-2 SWIR (1570–2290)-1 cirrus cloud detection (1360–1380)-2 TIRS (10,600–12,510)	30–15–100	170	16
	Landsat-7 ETM+	15 April 1999	6 (450–1750)-1 Pan (520–900)-1 (2090–2350)-1 (1040–1250)	30–15–60	183	16
	Landsat-5 TM	1 March 1984	5 (450–1750)-1 (2080–2350)-1 (1040–1250)	30–120	185	16
	Landsat-5 MSS	1 March 1984	4 (450–1750)-1 Pan (1040–1250)	80	185	18
	EO-1 Hyperion	21 November 2000	242 (350–2570)	30	7.5	16
	EO-1 ALI	21 November 2000	9(433–2350)-1 Pan (480–690)	10–30	185	16
	Terra ASTER	18 December 1999	3 VNIR (520–860)-6 SWIR (1600–2430)-5 TIR (8125–11,650)	15–30–90	60	16
	PROBA CHRIS	22 October 2001	19 in the VNIR range (400–1050)	18–36	14	7
	HICO	10 September 2009	128 (350–1080)	100	45–50	10
**Regional-Global Resolution**	Terra MODIS	18 December 1999	2 (620–876)-5 (459–2155)-29 (405–877 and thermal)	250–500–1000	2330	1–2
	Envisat-1 MERIS	1 March 2002	15 (390–1040)	300–1200	1150	daily
	OrbView-2 SeaWiFS	1 August 1997	8 (402–885)	1130	2806	16
	NIMBUS-7 CZCS	24 October 1978	6 (433–12,500)	825	1556	6
	ERS-1 ATSR-1	17 June 1991	1 SWIR (1600), 1 MWIR (3700), 2 TIR (10,850–12,000), Nadir-viewing Microwave Sounder with channels at 23.8 and 35.6 GHz	1000 (MW sounder: 20 km)	500	3–6
	ERS-2 ATSR-2	22 April 1995	3 VIS-NIR (555–865), 1 SWIR (1600), 1 MWIR (3700), 2 TIR (10,850–12,000)	1000	500	3–6
	ENVISAT AATSR	1 March 2002	3 VIS-NIR (555–865), 1 SWIR (1600), 1 MWIR (3700), TIR (10,850–12,000)	1000	500	3–6
	Suomi NPP VIIRS	28 October 2011	5 I-bands (640–1145), 16 M-bands (412–12,013), DNB (500–900)	375–750	3060	1–2 times a day
	NOAA-16 AVHRR	21 September 2000	6 (650–1230)	1100–4000	3000	9

**Table 2:** Specification of the more commonly used airborne sensors in water quality assessments.

Types of Sensors	Full Name	Manufacturer	Type	Scan System	Number of Bands	Spectral Range (μm)	Resolution (m)	Imaging Swath
**AVIRIS**	Airborne Visible Infrared Imaging Spectrometer	NASA Jet Propulsion Lab.	Hyperspectral	Whiskbroom	224	0.40–2.50	17	12 km and 614 pixels per scanline
**HYDICE**	Hyperspectral Digital Imagery Collection Experiment	Naval Research Lab.	Hyperspectral	Pushbroom	210	0.40–2.50	0.8 to 4	270 m at the lowest altitude
**HyMap**	in the U.S. known as PROBE-1	Earth Search Sciences Inc.	Hyperspectral	Whiskbroom	128	0.40–2.50	3 to 10	512 pixels
**APEX**	Airborne Prism Experiment	VITO (Belgium)	Hyperspectral	Pushbroom	Up to 300 VIS/NIR (114), SWIR (199)	VIS/NIR (0.38–0.97), SWIR1 (0.97–2.50)	2 to 5	2.5–5 km
**CASI-1500**	Compact Airborne Spectrographic Imager	ITRES Research Limited	Hyperspectral	Pushbroom	Up to 228	0.40–1.00	0.5 to 3	512 pixels per scanline
**EPS-H**	Environmental Protection System	Geophysical and Environmental Research Imaging Spectrometer	Hyperspectral	Whiskbroom	VIS/NIR (76), SWIR1 (32), SWIR2 (32), TIR (12)	VIS/NIR (0.43–1.05), SWIR1 (1.50–1.80), SWIR2 (2.00–2.50), TIR (8–12.50)	Dependent upon flight (minimum 1 m)	89°
**DAIS 7915**	Digital Airborne Imaging Spectrometer	GER Corporation	Hyperspectral	Whiskbroom	VIS/NIR (32), SWIR1 (8), SWIR2 (32), MIR (1), TIR (12)	VIS/NIR (0.43–1.05), SWIR1 (1.50–1.80), SWIR2 (2.00–2.50), MIR (3.00–5.00), TIR (8.70–12.30)	3 to 20 depending on altitude	512 pixels per scanline
**AISA**	Airborne Imaging Spectrometer	Spectral Imaging	Hyperspectral	Pushbroom	Up to 288	0.43–0.90	1	364 pixels per scanline
**MIVIS**	Multispectral Infrared and Visible Imaging Spectrometer	Daedalus Enterprise Inc., USA	Multispectral	Whiskbroom	102 VIS/NIR (28), MIR (64),TIR (10)	VIS (0.43–0.83), NIR (1.15–1.55), MIR (2.0–2.5) TIR (8.2–12.7)	3 to 8 depending on altitude	5.6 km at 4000 m altitude
**Daedalus**	Daedalus Multispectral Scanner (MSS)	Daedalus Enterprise Inc., USA	Multispectral	Pushbroom	12 VIS/NIR (8), SWIR (2), TIR (2)	0.42–14.00	25	714 pixels per scanline
**HySpex ODIN-1024**	HySpex hyperspectral cameras	Norsk Elektro Optikk (NEO)	Hyperspectral	Pushbroom	VIS/NIR1 (128), VIS/NIR2 (160), SWIR1 (160), SWIR2 (256)	0.40–2.50	0.5 m at 2000 m altitude	500 m

**Table 3:** Characteristics of the more commonly used microwave radiometers in oceanography and water quality studies.

Satellite	Sensor	Full Name	Launch	Failure	Frequency (GHz)	Spatial Resolution (km)	Swath Width (km)	Purpose
Nimbus-5	ESMR	Electrically Scanning Microwave Radiometer	December 1972	May 1977	19.4	25 for all Channels	3000	SST
Nimbus-7	SMMR	Scanning Multichannel Microwave Radiometer	October 1978	May 2015	6.6, 10.7, 18.0, 21.0, and 37.0	25 for all Channels	800	SST
SEASAT	SMMR	Scanning Multichannel Microwave Radiometer	June 1978	October 1978	6.6, 10.7, 18.0, 21.0, and 37.1	22 at 37.1 GHz to 100 at 6.6 GHz	600	SST
Priroda-MIR	IKAR-P	Ikarus Panoramic microwave radiometer	April 1996	March 2001	5.0, 13.3	75 for all Channels	750	SST
POEM-1	MIMR	Multifrequency Imaging Microwave Radiometer	June 1998	_	6.8, 10.7, 18.7, 23.8, 36.5, and 90.0	4.8 × 3.1 at 90 GHz to 60 × 40 at 6.8 GHz	1400	SST
EOS PM-1	MIMR	Multifrequency Imaging Microwave Radiometer	May 2002	Present	6.8, 10.7, 18.7, 23.8, 36.5, and 90.0	4.8 × 3.1 at 90 GHz to 60 × 40 at 6.8 GHz	1400	SST
TRMM	TMI	TRMM Microwave Imager	November 1997	April 2015	10.7, 19.4, 21.3, 37.0, and 85.5	8 × 6 at 85.5 GHz to 72 × 43 at 10.7 GHz	760	SST
ADEOS-2	AMSR	Advanced Microwave Scanning Radiometer	December 2002	October 2003	6.9, 10.7, 18.7, 23.8, 36.5, 50.2, 53.8, 89.0	6 × 3 at 89 GHz to 70 × 40 at 6.9 GHz	1600	SST
AQUA	AMSR-E	Advanced Microwave Scanning Radiometer for EOS	May 2002	October 2011	6.9, 10.7, 18.7, 23.8, 36.5, and 89.0	6 × 4 at 89.0 GHz to 75 × 43 at 6.9 GHz	1450	SST
GCOM-W1	AMSR-2	Advanced Microwave Scanning Radiometer—2	May 2012	Present	6.9, 7.3, 10.7, 18.7, 23.8, 36.5, and 89.0	5 × 3 at 89.0 GHz to 62 × 35 at 6.9 GHz	1450	SST
GPM	GMI	GPM Microwave Imager	July 2013	Present	10.7, 18.7, 23.8, 36.5, 89.0, 166.0, and 183.3	7.2 × 4.4 at 183.3 GHz to 32 × 19 at 10.7 GHz	850	SST
Coriolis	WindSat	WindSat	January 2003	Present	6.8, 10.7, 18.7, 23.8, and 37.0	13 × 8 at 6.8 GHz to 71 × 39 at 37.0 GHz	1000	SST
SAC-D	Aquarius	Aquarius	May 2011	June 2015	1.413	100 for all Channels	390	SSS-SST
SMOS	MIRAS	Microwave Imaging Radiometer using Aperture Synthesis	November 2009	October 2013	1.413	50 for all Channels	1000	SSS
Airborne	ESTAR	Electronically Scanning Thinned-Array Radiometer	Deployed in 1990	_	1.413	100 for all Channels	600	SSS
Airborne	PALS	Passive Active L- and S-band Sensor	Deployed in 1999	2009	1.413	0.350–1	16	SSS-SST
Airborne	2D-STAR	Two-Dimensional Electronically Scanning Thinned-Array Radiometer	Deployed in 2003	Present	1.413	0.800 for all Channels	10	SSS
Airborne	SLFMR	Scanning Low Frequency Microwave Radiometer	Deployed in August 1999	_	1.413	0.5–1	Twice the altitude	SSS
Airborne	STARRS	Airborne Salinity, Temperature, and Roughness Remote Scanner	Deployed in June 2001	_	**L-Band:** 1.413	1 for all Channels	5.2	SSS-SST
					**C-Band:** 5.2, 5.6, 5.9, 6.2, 6.6 and 7.1			
					**IR radiometer:** 8–14 and 9.6–11.5 micron			

**Table 4:** The most commonly measured qualitative parameters of water by means of remote sensing.

Water Quality Parameter	Abbreviation	Units	Optical Activity	References
chlorophyll-*a*	CHL-*a*	mg/L	Active	[10,36,37,38]
Secchi Disk Depth	SDD	m	Active	[39,40,41,42]
Temperature	T	°C	Active	[43,44,45,46]
Colored Dissolved Organic Matters	CDOM	mg/L	Active	[10,47,48,49]
Total Organic Carbon	TOC	mg/L	Active	[50,51,52]
Dissolved Organic Carbon	DOC	mg/L	Inactive	[53,54,55]
Total Suspended Matters	TSM	mg/L	Active	[56,57,58,59]
Turbidity	TUR	NTU	Active	[60,61,62]
Sea Surface Salinity	SSS	PSU	Active	[63,64,65,66]
Total Phosphorus	TP	mg/L	Inactive	[29,36,67,68,69]
Ortho-Phosphate	PO_4_	mg/L	Inactive	[70]
Chemical Oxygen Demand (COD)	COD	mg/L	Inactive	[71,72,73,74]
Biochemical Oxygen Demand	BOD	mg/L	Inactive	[62,75,76,77]
Electrical Conductivity	EC	µs/cm	Active	[78,79,80]
Ammonia Nitrogen	NH_3_-N	mg/L	Inactive	[73,81,82]

**Table 5:** Remotely measurements of chl-*a* using various spectral bands and their ratios.

Band Combination		Sensor	Reference
Ratio between green (0.50–0.60 μm) and red (0.60–0.70 μm)		Landsat 5-TM	[3,12,26,42,102,103,104,105,106]
		Landsat 5-MSS	[24]
		Landsat 7-ETM+	[107]
		SPOT	[108]
		IRS-LISS-III	[71]
Ratio between near infrared (NIR) and red		Landsat 5-TM	[109]
		HICO	[110,111,112]
		PROBA-CHRIS	[113]
		MODIS	[22,114,115]
		MERIS	[115,116,117]
		AISA	[115,118]
Ratio between green and blue (B2/B1)		Landsat 5-TM	[58]
		Landsat 7-ETM+	[119]
		MERIS	[120]
		PROBA-CHRIS	[121]
		EO-1 Hyperion	[122]
Ratio between blue (0.40–0.50 μm) and red (0.60–0.70 μm)		Landsat 5-TM	[26]
		Landsat 7-ETM+	[98]
Using a single band	Blue (0.40–0.50 μm)	Landsat 5-TM	[11,88,123,124]
	Red (0.60–0.70 μm)	PROBA-CHRIS	[125]
		Landsat 5-TM	[86]
		CASI	[126]
	Green (0.50–0.60 μm)	Landsat 5-TM	[127]
		Daedalus Airborne Thematic Mapper (ATM)	[87]

**Table 6:** Remotely measurements of CDOM using various spectral bands and their ratios.

Spectral Bands	Sensor	Reference
Single blue band (0.40–0.50 μm)	Landsat 5-TM	[42]
	EO-1 Hyperion	[122]
	SeaWiFS + MODIS-Aqua	[159]
	MODIS	[160,161]
	SeaWiFS	[48,162,163]
	HICO	[47]
	CZCS	[164]
Ratio between blue (0.40–0.50 μm) and green (0.50–0.60 μm)	ALOS-AVNIR-2	[56]
	MODIS	[165]
	SeaWiFS	[166,167,168,169]
Ratio between green (0.50–0.60 μm) and red (0.60–0.70 μm)	MODIS	[114]
	HICO	[112]
	EO-1 ALI	[146,170,171]
	EO-1 Hyperion	[49]
	SeaWiFS	[172]
	MERIS	[173]

**Table 7:** Remotely measurements of SDD using various spectral bands and their ratios.

Band Combination		Sensor	Reference
Ratio between blue (0.40–0.50 μm) and green (0.50–0.60 μm)		Landsat 5-TM	[27,190]
		Landsat 5-MSS	[192]
		Landsat 7-ETM+	[107]
		ASTER and ETM+	[197]
Ratio between blue (0.40–0.50 μm) and red (0.60–0.70 μm)		Landsat 5-TM	[39,42,60,102,179,186,198,199,200]
		Landsat 5-MSS	[24]
		PROBA-CHRIS	[121]
		IKONOS	[28,60]
Ratio between green (0.50–0.60 μm) and red (0.60–0.70 μm)		Landsat 5-TM	[30,194]
		ALOS-AVNIR-2	[56]
		SPOT	[108,201]
Using a single band	Blue (0.40–0.50 μm)	Landsat 5-TM	[69]
		MODIS	[41]
	Red (0.60–0.70 μm)	Landsat 5-TM	[12,30]
	Green (0.50–0.60 μm)	Landsat 5- MSS	[191]
		MODIS	[114]

**Table 8:** Remotely measurements of Turbidity and Total Suspended Sediments using various spectral bands and their ratios.

Band Combination		Sensor	Reference
Ratio between green (0.50–0.60 μm) and red (0.60–0.70 μm)		Landsat 5-TM	[30,213]
		PROBA-CHRIS	[121]
		IRS-LISS-III	[71]
Ratio between blue (0.40–0.50 μm) and red (0.60–0.70 μm)		Landsat 5-TM	[198]
		AISA	[22]
Ratio between near infrared (NIR) and red (0.60–0.70 μm)		MODIS	[59]
		ALOS-AVNIR-2	[56]
Using a single band	Near Infrared (0.75–0.90 μm)	SPOT	[215]
		Landsat 7- ETM+	[57]
		CASI	[29]
	Red (0.60–0.70 μm)	Landsat 7- ETM+	[216]
		Landsat 5-TM	[12,42,216]
		HICO	[112]
		PROBA-CHRIS	[216]
	Green (0.50–0.60 μm)	Landsat 5- MSS	[192]
		IRS-LISS-III	[217]

**Table 9:** Remotely measurements of total phosphorus (TP) using various sensors and blue and green bands, and integration of red and green bands ratio.

Band Combination	Sensor	Reference
Blue (0.45–0.51 μm) and green (0.50–0.60 μm) bands, and integration of red (0.60–0.70 μm) and green (0.50–0.60 μm) bands	Landsat 5-TM	[69]
	MODIS	[234]
	PROBA-CHRIS	[121]
	CASI	[29]
	SPOT	[108]

**Table 10:** Infrared thermal band applications to quantify the water temperature.

Sensor	Reference
TIR band of Landsat sensors (TM, ETM+, and OLI/TIRS)	TM: [43,198,255,256,257], ETM+: [43,45,197,253,258,259,260,261], OLI/TIRS: [46,262,263]
TIR band of MODIS	[22,41,45,264,265]
TIR band of ASTER	[45,197,253,260,261,266]
TIR band of AVHRR	[44,267,268,269,270]
TIR band of airborne MODIS/ASTER (MASTER)	[45,253,261,266]
Sea Surface Temperature monitoring studies using microwave radiometers (MWRs)	WindSat: [271,272], AATSR: [273,274,275,276], ATSR-1: [277,278,279], ATSR-2: [273,280,281,282], AMSR-E: [283,284], TMI: [285,286,287]

**Table 11:** Remote sensing of Sea Surface Salinity (SSS) based on the used sensor.

Sensor	Reference
European Soil Moisture and Ocean Salinity (SMOS)	[63,65,289,293,307,308,309,310,311,312,313]
Aquarius L-band radiometer carried by the SAC-D	[66,314,315,316]
SLFMR	[300,317,318,319,320]
STARRS	[301,319,321]
Other MWRs experiences	PALS: [322,323,324], AMSR-E: [325] 2D-STAR and ESTAR: [326,327,328]
predicted indirectly by making relationship between salinity and temperature	[64,288,294,295,297,298,299,300,301,329]
predicted indirectly by making relationship between salinity and CDOM	[54,166,302,303]

**Table 12:** Remote sensing of dissolved oxygen (DO), biochemical oxygen demand (BOD), and chemical oxygen demand (COD) based on the used sensor.

Sensor	Reference
Landsat 5-TM	[61,73,74,77]
Landsat 5-MSS	[75]
WorldView-2	[9]
IRS-LISS-III	[71]
MODIS	[169]
MERIS	[169]
AVHRR	[62]
SeaWiFS	[236]
SPOT	[76]

**Table 13:** Comparison between spaceborne and airborne sensors.

Parameter	Spaceborne	Airborne
Time of overpass	Mostly fixed	Flexible
Spatial resolution	Ground Sampling Distance (GSD) up to 0.5 m for panchromatic images. For multi-band images, it ranges from a few meters (low altitude sensors) up to a few kilometers for high altitude sensors	Ground Sampling Distance (GSD) < 5 m
Spectral resolution	Mostly panchromatic (one band) to multispectral, recently developed sensors like HyspIRI, CHRIS, and HICO are hyperspectral	Panchromatic to hyperspectral
Temporal resolution (Revisit time)	Days	Minutes
Calibration	Precalibration before launch, then on-board characterization (usually yearly)	Before launch + possible on-board
Cost	Free (non-commercial), up to about $50 per sq km (commercial). High spatial resolution imagery can be very expensive (~$2–10 k per scene)	Average costs of $350 per square mile (Chipman et al. 2009)
Stability	High	Low, due to turbulence
Swath width	High (up to 2500 km for low altitude sensors, a full hemisphere for high altitude sensors)	Small (up to 10 km per flight line)
Interpretation approaches	Mostly empirical-and semi-empirical-based approaches	Both empirical and analytical approaches
Complexity of image processing	Less complex compared to hyperspectral sensors	Processing of hyperspectral images is more complex and requires specific skills
Constraints	Limited to the coverage schedule of the satellite, including weather/cloud constraints; this can be challenging when trying to conduct water quality monitoring at a certain time of the year or dealing with project schedules	Coverage schedule is flexible
Geographic coverage areas	Local, regional, and global	Local and regional
